# Mortality trends in pulmonary arterial hypertension: Are we just spinning our wheels?

**DOI:** 10.1002/pul2.12162

**Published:** 2022-10-01

**Authors:** Jason G. E. Zelt, Jason Weatherald

**Affiliations:** ^1^ Department of Medicine, Faculty of Medicine University of Ottawa Ottawa Canada; ^2^ Department of Medicine, Division of Pulmonary Medicine University of Alberta Edmonton Canada

**Keywords:** mortality, pulmonary arterial hypertension

Pulmonary arterial hypertension (PAH) is a progressive vasculopathy of the distal pulmonary arterioles that remains an incurable disease with a poor prognosis. The past few decades have witnessed steady progress in the classification, diagnosis, and clinical management of patients with PAH. This evolution in management has now been reflected in four iterations of the European Cardiology Society/European Respiratory Society pulmonary hypertension clinical guidelines. The introduction of epoprostenol in the late 90s revolutionized the management for patients with PAH, transitioning management from supportive care alone to one with disease‐modifying potential. There has also been a trend toward consolidation of care in PH centers where experts have access to an expanding arsenal of medications that predominantly work by vasodilation, target three aberrant pathways: prostacyclin, endothelin‐1, and nitric oxide. Over time, there has been a movement to more aggressive upfront management with dual or even triple therapy for newly diagnosed patients, and a sequential add‐on strategy for prevalent patients not meeting treatment goals.^1^ The diagnostic criteria for PAH have also become more liberal to reflect emerging data of hemodynamic risk with milder elevation in pulmonary artery pressure and pulmonary vascular resistance, which may permit earlier identification of the disease. With all these changes, it is important to assess outcomes/disease metrics to ensure these changes are routed in sound scientific rationale, but also translate to meaningful clinical impact.

In this issue of Pulmonary Circulation, Hendriks et al.^2^ studied secular trends in PAH characteristics at diagnosis, treatment strategies, and mortality in the Netherlands at two reference centers. They described an increase in the use of initial and sequential combination therapy after publication of the 2015 ESC/ERS guidelines which was not paralleled with improved transplant‐free survival. Surprisingly, despite a tendency to having less severe hemodynamics at diagnosis, they found worse survival in patients diagnosed 2015−2019 as compared to 2005−2009, which authors hypothesize may result from the ~50% decrease in the use of prostacyclin analogues. Although provocative, these findings need to be interpreted cautiously given relatively small sample sizes in their study, particularly in the group diagnosed in 2005−2009 (*n* = 56). Furthermore, there was a higher proportion with underlying connective tissues diseases, specifically systemic sclerosis, in the more recent era, which is a group known to have worse survival compared to idiopathic PAH patients.

This study is important in light of three other recent publications that evaluated temporal changes in survival in PAH. We recently performed a multicentre retrospective cohort study in Canada on survival trends in PAH mortality across two iterations of the ESC/ERS clinical guidelines from 2009 to 2020.^3^ Our aim was to assess whether changes in management, as reflected in changing guidelines, translated to improved long‐term survival for patients with PAH. In this Canadian cohort, the 1‐, 3,‐ and 5‐year survival rates were 89.2%, 75.6%, and 56.0%, respectively. This is nearly identical to the transplant‐free survival in the Dutch study by Hendriks et al. We found no significant differences in 1‐year (88.8% vs. 89.5%) or 5‐year survival (53.8% vs. 58.9%) before and after publication of the 2015 ESC/ERS guidelines despite a clear increase in upfront or sequential combination therapy. At the same time, Hoeper et al.^4^ replicated these findings in the COMPERA registry comprising >2500 incident and treatment naïve PAH patients. More recently, in a single‐center cohort of systemic sclerosis‐associated PAH out of John Hopkins found that patients diagnosed from 2010 to 2021, compared to 1999−2010 had a increased median transplant‐free survival by 4 years.^5^ While a survival improvement for this systemic sclerosis‐associated PAH subgroup is optimistic and encouraging, analyses of systemic sclerosis patients over such a long period may be prone to lead‐time bias, as active screening for PAH in systemic sclerosis patients also likely increased during their study duration. In keeping with this assertion, clinical and hemodynamic characteristics were less severe at diagnosis in patients diagnosed after 2010, though the use of combination therapy also increased.

The advent of combination therapy has yielded undisputable clinical benefit to patients as demonstrated in several large RCTs.^6^, ^7^, ^8^ Meta‐analyses of these trials have demonstrated an overall benefit of combination therapy on time to clinical worsening, but have produced mixed findings on mortality.^6^, ^7^ Emerging real‐world data from contemporary clinical registries may shed light on the impact of sequential or upfront combination therapy on long‐term survival. In the Canadian cohort, there was no difference in survival between patients placed on initial mono versus dual therapy, even after adjusting for baseline mortality risk and disease severity.^3^ Early data from the Pulmonary Hypertension Association Registry showed that initial combination therapy (dual and triple) was associated with a lower 1‐year mortality, but no difference in year 2 or 3‐year mortality.^9^ The French PAH Registry also found no difference in long‐term survival between those treated with upfront mono or dual therapy; however, triple therapy including parenteral prostacyclin improved 5‐year mortality.^10^ Historically, intravenous epoprostenol is the only PAH therapy that a demonstrated survival benefit in a single short‐term multicentre randomized trial.^11^ This data may add credence to the Hendriks et al. hypothesis of worsening survival over time, mediated by decreased parenteral prostacyclin use. Taken together, these data suggest that contemporary management may not have ultimately improved long‐term survival in real world PAH populations (which differ from PAH clinical trial populations). Though the strategy of initial triple oral therapy with selexipag, macitentan, and tadalafil was not proven to be superior to dual oral therapy in treatment naïve patients,^12^ compelling observational data suggest that more aggressive right ventricular afterload reduction with triple therapy that includes a parenteral prostanoid might improve long‐term prognosis.^10^ These studies of temporal trends in mortality are a reality check and seem to conflict with our perception of progress made in the field of PAH. In the Disney movie, *The Lion King*, a wise character proclaims, “the past can hurt, but the way I see it, you can either run from it, or learn from it.” While we await new therapies targeting novel pathways that modify the prognosis of PAH, more data validating the utility of aggressive upfront treatment with parenteral therapies would be useful as we continue to battle this devastating disease in an era without a cure.

## AUTHOR CONTRIBUTIONS

Jason G. E. Zelt and Jason Weatherald conceived and wrote the manuscript.

## CONFLICTS OF INTEREST

Jason Weatherald has received research grants paid to his institution from Janssen, Actelion, Merck, and Bayer; consulting fees paid personally from Janssen; honoraria for lectures from Janssen; payment for expert testimony from Sprigings Intellectual Property Law; travel support from Janssen, and payment for participation in advisory boards from Janssen and Acceleron. The remaining author declare no conflict of interest.
